# Strategies for Integrated Analysis of Genetic, Epigenetic, and Gene Expression Variation in Cancer: Addressing the Challenges

**DOI:** 10.3389/fgene.2016.00002

**Published:** 2016-02-01

**Authors:** Louise B. Thingholm, Lars Andersen, Enes Makalic, Melissa C. Southey, Mads Thomassen, Lise Lotte Hansen

**Affiliations:** ^1^Department of Pathology, The University of MelbourneMelbourne, VIC, Australia; ^2^Department of Biomedicine, The University of AarhusAarhus, Denmark; ^3^Department of Clinical Genetics, Odense University HospitalOdense, Denmark; ^4^Centre for Epidemiology and Biostatistics, The University of MelbourneMelbourne, VIC, Australia

**Keywords:** integrated analysis, array data, massive parallel sequencing (MPS), DNA methylation, gene expression

## Abstract

The development and progression of cancer, a collection of diseases with complex genetic architectures, is facilitated by the interplay of multiple etiological factors. This complexity challenges the traditional single-platform study design and calls for an integrated approach to data analysis. However, integration of heterogeneous measurements of biological variation is a non-trivial exercise due to the diversity of the human genome and the variety of output data formats and genome coverage obtained from the commonly used molecular platforms. This review article will provide an introduction to integration strategies used for analyzing genetic risk factors for cancer. We critically examine the ability of these strategies to handle the complexity of the human genome and also accommodate information about the biological and functional interactions between the elements that have been measured—making the assessment of disease risk against a composite genomic factor possible. The focus of this review is to provide an overview and introduction to the main strategies and to discuss where there is a need for further development.

## Introduction

Aberrant function of proteins and changes in gene expression are central elements of disease onset and progression. A key focus of genetic research is to identify the molecular aberration(s) that “cause” and promote disease development. However, the size and complexity of the human genome, combined with the epigenome, renders the identification and interpretation of genetic findings very difficult, time consuming, and computationally intense.

Many types of variations, both genetic and epigenetic, have been identified that can disrupt gene function. Key examples of variations that can impact gene function include, gene copy number (CN), DNA methylation, single nucleotide variations (SNV), and indels (small insertions and deletions). When located in the coding region of genes CNs, SNVs and indels can alter the function of the gene product and when located in untranslated regions (UTRs) these variations can interfere with gene expression by inhibiting transcription. Non-coding RNAs exhibit a regulatory function on target genes causing alternations in transcribed RNAs and thereby indirectly influence gene expression. As molecular methods become more sophisticated an increasing number and variety of molecular variants are being described and associated with disease susceptibility (Patch et al., 2015).

Recent technological developments have enabled the creation of genome-wide data for multiple types of variations. Each dataset generally provides information about one type of variation, and singular analyses of these datasets have led to our understanding of a long list of single gene disorders. However, with increasing knowledge of the genome and of complex disorders, it is becoming clear that isolated analyses of the different types of variations may only provide a linear view of a multidimensional landscape. Therefore, integrated analysis is necessary to reach in-depth understanding of common disorders and measure the possible interactions of risk factors identified in the linear, yet often genome-wide, analyses.

The number of studies in which integration has been successfully applied, as well as the number of tools developed to facilitate the integration is rapidly increasing. The goal of this review is to provide an overview of:

1. The main integration strategies for identification of genetic variation functionally relevant to cancer susceptibility and progression, together with an evaluation of the strategies biological and statistical limitations.
2. The main applications of the integration strategies in cancer research addressing somatic and heritable genetic variations.

Data integration can be divided into two main categories (a) integrated analysis of data with information on one type of variation, such as the integration of expression data generated in different studies (single-platform integration), and (b) integrated analysis of data with information on different types of variation such as integrated analysis of expression and methylation data (cross-platform integration). A similar distinction was introduced previously by Hamid et al. (2009).

Combining similar data sets generated in different laboratories, at different times or on different versions of a platform, is common and referred to as meta-studies (combining data by using the summary statistics) or mega-studies (combining raw data). The advantages of meta-studies have led to the development of a number of statistical models (Hong and Breitling, 2008; Natarajan et al., 2012; Evangelou and Ioannidis, 2013). This review will address cross-platform integration [category (b) above] due to the potential these approaches have to significantly improve our understanding of complex diseases such as cancer.

## Cancer and data integration

In the context of cancer research, it is important to distinguish between the issues related to the analysis of tumor-derived and non-tumor-derived (e.g., blood-derived) data. Tumor genomes are usually highly altered comprising both driver mutations and passenger mutations caused by an increased genomic instability. As a consequence, the aim of studies working with the integration of tumor-derived data differs from the aim of studies focusing on heritable risk factors. Studies of data from tumors often aim to identify driver mutations among the long lists of somatic mutations, whereas studies focusing on inheritance of cancer risk aim to identify one or a few disease associated mutation in cancer susceptibility genes. Examples of strategies and tools for integrated analysis of heritable cancer-causing variations are few. However, examples of data integration exist for the analysis of heritable risk factors for other complex disorders such as asthma and as these methods could inspire development of methods for cancer we will in the discussion of heritable risk factors include methods used in the context of other diseases.

The complex genetic architecture associated with common disorders such as cancer complicate the identification of disease-associated genetic variations. A high level of allelic and locus heterogeneity are some of the factors associated with complex disorders (McClellan et al., 2007). This heterogeneity can cause diseases with similar clinical features to be associated with different genetic and epigenetic variations across cases. Multiple etiological disease-associated factors may be, (1) co-located resulting in an additive or inter-dependent effect or (2) found at specific loci and cause a high level of allelic heterogeneity across affected individuals. The success of cross-platform integration studies for identification of genetic locations involved in cancer susceptibility is therefore highly dependent on the ability of the statistical method to identify rare, perhaps case specific variations.

Ideally, the analysis method should be sensitive to an additive or multiplicative influence from low effect events and at the same time be capable of identifying variants of very low frequency associated with high risk. For the analysis to be successful the data must be specific for each individual in the sample set, which means that each type of variation can be identified, if present, for each individual. This presents a statistical challenge in situations where the aim is to identify a difference in a continuous variable/measurement between cases and controls (such as for DNA methylation or gene expression).

For gene expression data, commonly collected in studies of tumor genomes, this challenge is often met by calculating a mean level across controls if such are available, and then identify the extent to which the gene expression level in each case deviates from the mean. If no control data is available, a mean expression level or gene expression distribution can be calculated across all cases to which the individual case can be compared. The hypothesis supporting this approach is that each gene/transcript is expected to only “cause” cancer in a subset of the cases in the sample set. Many different approaches can be used depending on the research question being addressed. For example, Gevaert et al. (2013) used disease-specific genomic analysis (DSGA) (Nicolau et al., 2007) to model disease-specific gene expression for the AMARETTO (Gevaert et al., 2013) tool, and MethylMix has been developed to identify genes that are significantly differentially methylated in a subset of cases in the dataset when compared to normal tissue (Gevaert, 2015). Further aspects of these tools are discussed below.

Basic principles of cancer tumorigenic models may be implemented in the identification of driver genes. Classically, cancer driver genes are divided into tumor suppressor genes (TSG) and oncogenes. Tumor suppressor genes are required to prevent uncontrolled cell growth and according to the classical two hit hypothesis (Knudson, 1971) both copies of a TSG needs to be inactivated for a cancer to develop. In hereditary cancer an inherited mutation in a tumor suppressor gene is often followed by loss of the wildtype allele via somatic mutation. Both first and second hit mutations are expected to be inactivating mutations (Vogelstein and Kinzler, 1992). Contradictorily, an oncogene is the activated form of a proto-oncogene that typically code for genes involved in growth factor signaling pathways (Huebner and Todaro, 1969). The mutations activate oncogenes, i.e., mutations in active sites, regulatory regions or via gene amplification. A classical example is the fusion of the BCR gene on chromosome 22, regulated by a constitutive promotor, with the ABL kinase gene involved in intracellular secondary message of growth factor signaling on chromosome 9, causal for chronic myelogenous leukemia (Heisterkamp et al., 1985). These principles are to some extend implemented in the strategies discussed in the following sections.

To summarize, research into the genetic landscape of cancer distinguish between tumor genomes and non-tumor genomes. Both settings are characterized by a high level of genetic complexity including different types of variations that can be rare or common with high or low effects. The mechanism by which the genetic variations contribute to disease development and progression differs between the two settings and current strategies show how knowledge of the different mechanisms can support development of tools for the identification of relevant variations.

## Identification of driver genes in tumor-derived data sets

A large fraction of today's cross-platform integration projects analyze tumor genomes with the aim of identifying driver genes. Some general strategies can be observed in these studies, depending on the type of tissue and measurements that are available and the level of genetic complexity that the study aims to consider. In the simplest situation, studies evaluate the presence of different types of variations at the same genetic location, e.g., when a copy number deletion in gene A is found to be associated with a down-regulation of gene A expression.

As an in-sequence variation must have a functional effect in order to lead to disease, many studies integrate in-sequence data with gene expression data, arguing that if an in-sequence variation leads to a change in expression of a co-located or distant gene, the variation is more likely to be disease relevant. TSGs are expected to have a decreased function, while oncogenes are expected to have an increased function. Addou-Klouche et al. used this theory to identify TSGs by identifying genes harboring CNVs and showing that these tumors have a decreased expression level of the gene as compared to tumors not containing the CNV (Addou-Klouche et al., 2010). The difference in expression levels between the two groups is tested with a student *t*-test. A similar approach can be used for the identification of oncogenes by identifying CNVs associated with increases in gene expression.

Another integration strategy based on co-location of variations for identification of drivers is the identification of overlapping hits between the two or more datasets. First, each variation type is analyzed separately to identify a ranked list of affected genes. The data-specific analysis and filtering performed to obtain these lists can vary greatly, which is a strength in the aspect that most types of data can be used, but when pre-integration analysis and filtering is not supported by the larger amount of information available in the additional datasets possible integration-dependent discoveries can be lost. In these instances data is integrated by the identification of genes present on both the lists, and results are often illustrated using Venn diagrams. For example, Hassan et al. uses a list intersection-based strategy to identify genes showing both differential expression and CNV between colorectal cancer samples and non-cancerous tissue (Ali Hassan et al., 2014).

An increasing number of studies include both CNV and mutations (SNPs and indels) in integration studies. The motivation here is the anticipation that multiple types of genetic variation can either be the cause of, or contribute to, the same phenotype. If a given gene or pathway is altered at a high frequency across modalities, but at a low frequency for any one modality, it is likely that the gene/pathway would be overlooked in single-variation analysis. In this setting, the number of cases whose causative variation is included in the analysis increases when multiple modalities are considered.

Leary et al. integrated CNV and mutation data for breast and colorectal cancers by a co-location approach. Datasets were pre-analyzed to obtain a list of variant-containing genes for each data type (Leary et al., 2008). They then distinguished between drivers and passengers by utilizing the theory that drivers will be affected by in-sequence variation at a higher frequency than passenger genes. For each type of variation they calculated the probability that a gene was a driver gene by comparing observed mutation frequencies with mutation frequencies expected for passenger mutations. This probability was calculated for each gene for each type of variation and integrated, thereby identifying possible driver genes and pathways.

Ding et al. (2008) also utilizes the assumption that drivers contain variation at a higher frequency than expected by chance (Wood et al., 2007) for analysis of data derived from lung adenocarcinomas. Here, different types of variations were not integrated for the identification of possible drivers but the study provides a thorough discussion about the consequences of using different approaches to calculate driver probabilities. Following identification of possible driver mutations, the mutations were further evaluated by analyzing their co-location with CNVs and their correlation (Pearson) with CNV and gene expression, again basing the integration on co-location.

DNA methylation is known to affect gene expression and has been associated with tumor development. It is therefore relevant to include DNA methylation in studies aiming to identify the full set of driver genes in tumor-derived datasets. Wrzeszczynski et al. analyzed CNV, gene expression and DNA methylation data derived from ovarian tumors (Wrzeszczynski et al., 2011). One of their many analyses aimed to identify TSG and oncogenes affected by all three types of variations. For genes affected by CNV they calculate DNA methylation and gene expression levels and identified TSGs as genes with CN deletion, increased methylation and decreased expression. Oncogenes were identified as amplified genes with decreased DNA methylation and increased expression. They further defined a possible regulatory feature exercised by DNA methylation to regulate gene expression of drivers affected by CNVs and identified genes for which CN amplification appears to be overruled by increased methylation resulting in decreased gene expression. They provide a good example of how the many regulatory possibilities complicate the integrated analysis and how the analysis would be further complicated by including regulatory features that can differ between individual tumor samples.

Chari et al. has argued that the power to detect disrupted genes and pathways increase when multiple types of variations are analyzed (Chari et al., 2010). Showing how integrated analysis of structural variation and DNA methylation changes in tumor samples allowed for detection of nearly five times as many disrupted genes in more than 50% of samples as compared to analysis of each variation type separately. Their results illustrate the greater sensitivity of an integrated approach thereby allowing for detection of disrupted genes that would be missed by single-platform analysis because the frequency or effect of each type of disruption is too low to be detected. This example also highlights the pressing need for proper approaches to correct for multiple testing and FDR when performing integration studies.

Strategies discussed so far in this section are built on co-location of variations for integration and identification of drivers. An overview of strategies can be found in Table 1. When a number of potential drivers are identified, the next step for many studies is to evaluate a potential connection between the genes in the form of shared pathways or networks (Ding et al., 2008; Wrzeszczynski et al., 2011). For evaluation of possible higher-level interaction different enrichment approaches are popular and easy-to-use online tools are becoming increasingly available.

**Table 1:** **Overview of integration strategies based on co-location of variations**.

**Integration strategies based on co-location of variations**				
**In-sequence variation**	**Supporting data**	**Integration strategies**		**Example study and tool if available**
CNV	Gene expression	Identification of CNV affected genes, followed by identification of a functional effect in the form of a change in gene expression of the co-located gene. Select genes by:	• Difference in mean expression between variant containing and not-containing tumors.	(Addou-Klouche et al., 2010) CMDD for identification of candidate genes (Ping et al., 2015)
			• Intersecting gene lists (Venn diagram).	Kikuchi et al., 2013; Ali Hassan et al., 2014
			• Correlation analysis of CNV and expression values.	
	DNA methylation and gene expression	Specification of modality-patterns expected for TSG and oncogenes. Integration on the basis of co-location and identification of genes showing specified pattern.		Wrzeszczynski et al., 2011
		Using a linear model for the effect of methylation and CNV on disease specific expression to identify potential drivers.		AMMERETTO 1. step (Gevaert et al., 2013)
Mutation	Gene expression and CNV	Identify a functional effect in the form of correlation with gene expression or CN of co-located gene.		Ding et al., 2008
CNV and mutation		Integration of variation types by co-location and selecting of drivers by theory of frequency.		Leary et al., 2008

Alternatively, strategies can aim to directly implement the interaction between genetic locations in the identification of drivers. An example scenario is that a mutation in location A co-occurs with a change in expression of gene B located at a distant genomic location. Such studies can implement prior knowledge of the interaction between genetic locations or can aim to identify novel interactions. The interactions considered can be the one-to-one as with gene A regulating gene B or at the higher pattern level of pathways or networks.

Masica et al. have developed a multistep workflow to identify genes with a mutation status that associates with the expression of genes that are not necessarily co-located (Masica and Karchin, 2011). The mutation data is pre-analyzed to the gene level resulting in a binary table of samples vs. genes with cells specifying if the gene is mutated in the sample or not. The expression data is similarly analyzed to the gene level and a table with relative expression of each gene for each sample is produced. Pairs of mutation-expression correlated genes are identified through a number of steps repeated for each gene in the mutation table. The process includes a 2-class, unpaired Significance Analysis of Microarrays (SAM) (Tusher et al., 2001) which uses a *t*-test to measure significance of expression between groups defined by the “response variable”—in this study the mutation status. Then genes from the expression table identified in the SAM step are converted to two binary matrices, one for overexpression and one for underexpression. Fisher's exact P value is calculated for a 2 × 2 contingency table (containing the binary expression and mutation data) to further filter the list of genes. The process is well explained and easy to follow including a number of corrections for multiple testing and false discovery rates. However, no code or tool is published to facilitate easy implementation of the strategy. The simple form of the mutation table does allow for easy extension to use the strategy on more types of variations e.g., CNV data.

Bashashati et al. have presented the R package DriverNet, developed for analyzing mutation, CNV and expression data (Bashashati et al., 2012). The aim of the tool is to identify functionally important drivers, where functional importance is seen as the number of connections a gene has to genes with outlying expression. The connections are based on prior knowledge of gene pathways obtained from Reactome (http://www.reactome.org/). An influence graph based on the network information is used to connect the variation types and a greedy algorithm finds the lowest number of genes connected to the most genes with outlying expression. Outlying expression of a gene in a sample is defined as the extremes of the expression distribution for the gene across all samples. Data on all variations considered to possibly affect expression levels is simplified to a binary matrix with samples as columns and genes as rows and with cells containing 1 if the gene contains variation in the patient or 0 if no variation is found. This requires a high level of preprocessing and does not consider possible interactions between the variations themselves or any additive effects. However, it is easy to include additional variation types in the analysis such as DNA mutation data.

DriverNet belongs to a group of tools built on the assumption that driver genes affect the expression of gene modules to a higher extend than single genes, and thereby on the underlying assumption that driver genes affect gene expression. The tools deviate in the types of expression-affecting variations they analyze and in their analytical approach. The strategy often depends on the assumptions of driver properties. CONEXIC assumes, in addition to the already stated assumptions, that driver mutations occur in multiple tumors more often than can be expected by chance (Akavia et al., 2010). It scores genes located in CNV regions in a significant number of samples based on how well they explain expression patterns of gene modules across tumors. Input data is gene expression levels and discrete CN values (normal, amplified, or deleted) for genes. The software is available online (www.c2b2.columbia.edu/danapeerlab/html/software.html).

AMARETTO developed by Gevaert et al. goes through two steps to identify drivers and their potential targets (Gevaert et al., 2013). The strategy implements CNV, DNA methylation and gene expression data. As it requires disease associated gene expression and DNA methylation it is necessary to have data from tumor and normal tissue. The first step uses a linear model for the effect of methylation and CNV on disease specific expression to identify potential drivers. Step two identifies target genes for the identified drivers by first identifying clusters of co-expressed genes and then applying a linear regression with elastic net regularization to select the drivers best regulating the expression module.

CaMoDi is a recent example of using the expression module approach to identify cancer drivers from expression data and potential regulators selected based on biological criteria from a database (Manolakos et al., 2014). As such, it is not an integration tool, however if potential regulator genes are selected from an available dataset, e.g., as all potentially damaging mutations in a massive parallel sequencing (MPS) dataset, the tool can be compared to AMARETTO and CONNEXIN as an integration tool.

The “expression module” approaches reviewed so far, have all identified modules directly from the available datasets. OncoImpact is an example where prior knowledge of interactions between genes (networks) is used (Bertrand et al., 2015). The tool also differs from the above approaches by aiming to identify tumor-specific drivers. An overview of strategies for integration based on “expression modules” or interactions between non co-located variations is found in Table 2.

**Table 2:** **Overview of strategies for integration based on expression modules or interactions of non-co-located variations**.

**Integration strategies based on “expression modules” or interactions of non-co-located variations**			
**In-sequence variation**	**Supporting data**	**Integration strategies**	**Example study and tool if available**
CNV	Gene expression	Identification of drivers as genes regulating expression modules.	CONEXIC (Akavia et al., 2010)
	Gene expression and DNA methylation	Step 1: identification of drivers as genes with significant relationship between genomic/epigenomic event and expression.	AMARETTO (Gevaert et al., 2013)
		Step 2: Identification of target modules for drivers.	
Mutation	Gene expression	Identification of genes with mutation status correlating with expression of other genes. Describes a workflow incl. SAM that compares all possible pairs of genes with data available.	Masica and Karchin, 2011
		Identification of drivers as genes regulating expression modules. Note that potential drivers are selected from a database and not included datasets.	CaMoDi (Manolakos et al., 2014)
CNV and mutation	Gene expression	Identification of drivers as genes regulating expression modules.	DriverNet (Bashashati et al., 2012)
			OncoImpact (Bertrand et al., 2015)

The above “expression module” based strategies aim to identify individual drivers. An alternative approach can be to identify sets of genes that show driver potential. Zhang et al. (2013) presented an approach that searches for “mutated core modules in cancer” as being gene sets contributing to cancer formation. The approach uses no prior knowledge of gene networks but constructs two weighted networks directly on the basis of the data. CNV and mutation data is combined to generate the first network and gene expression data is used to generate the second network. The two networks are combined to identify the most coherent sub-networks. From these, core modules are identified through further filtering including an exclusivity test based on an assumption that driver genes are not expected to co-occur in samples. The method takes into account a number of biological considerations regarding expected behavior of driver genes facilitating biologically relevant filtering. The method utilizes MPS data at the gene level with binary information (gene A in a DNA sample contains an in-sequence variation or no such variation). To obtain this format a high level of filtering and selection goes before the integration, as is most often seen when MPS data is included in integration studies.

Ping et al. have presented an alternative approach (CMDD) for the identification of “driver modules” and their target genes (Ping et al., 2015). This approach includes prior knowledge of gene interactions for clustering of candidate drivers and uses a linear regression model for selection of variations that affect gene expression levels.

In addition to the identification of driver genes, many cross-platform integration projects analyzing tumor-derived data aim to confirm cancer subtypes, and to more completely characterize the molecular architecture of each subtype (Nigro et al., 2005; Sun et al., 2011; Cancer Genome Atlas, 2012; Rakosy et al., 2013; Rhee et al., 2013). Integration with the goal of identifying subtypes is not the focus of this review, however an approach to stratify tumors by variation profiles in order to decrease the heterogeneity of the sample set, and in turn improve the ability to identify disease relevant variations is of indirect relevance to this discussion.

Tools for tumor stratification are not included in the review, but the improved stratification achievable through integration is highlighted by the following example. iCluster (Shen et al., 2009) was used to perform integrated analysis of molecular subtypes in Glioblastoma (Shen et al., 2012). This software tool allows for integrated analysis of multiple datasets from potentially different platforms and is clearly more efficient than manual comparison of clusters obtained from different analyses since much information is lost when performing cluster-analysis followed by manual data integration. Shen et al. (2012) integrated all array data from genome-wide DNA copy number, DNA methylation and gene expression data from “The Cancer Genome Atlas” (http://cancergenome.nih.gov) and successfully obtained new information on tumor subtypes which was not possible using any single analysis approach.

The section on integration strategies for identification of drivers in tumor-derived data highlights how strategies differ by the level of genetic complexity they aim to consider. As a result, most strategies can be roughly separated into two groups, (1) strategies that evaluate different types of variations at the same genetic location, and (2) strategies that implement the interaction between genetic locations. Strategies in the second group can implement prior knowledge of the interactions or can aim to identify novel interactions. The strategies can further be distinguished by whether they aim to identify individual drivers or “driver modules” as sets of genes that show driver potential.

Many integration strategies implement theory of the biological function of driver genes, e.g., to distinguish between drivers and passengers. TSGs are expected to have a decreased function, while oncogenes are expected to have an increased function. This theory is often used for the integration of expression data as a measure of function and to facilitates the integration of methylation data by using the theory of its effect on gene expression.

A common challenge for many strategies is to balance usability and the level to which they can consider complex and detailed genetic function e.g., by including more information from the different platforms. Often complex information obtained from e.g., MPS platforms is reduced to a simple format. To obtain this format a high level of filtering and analysis goes before the integration, highlighting both a need to thoroughly consider the quality of the data included in the integration and a limitation of current integration strategies.

## Identification of cancer risk loci

Genetic risk factors for complex disorders have long been analyzed in GWAS, and it is therefore not surprising that most integration strategies for complex disorders are built on ideas from GWAS or directly including “hits” from GWAS studies. Few integration studies exist for the identification of genetic risk factors for cancer. Therefore, we will discuss some published integration strategies for the identification of heritable genetic risk factors of complex diseases, which could play an important role in cancer research.

As for integrative studies of tumor tissue, gene expression data plays a significant role in integration studies for heritability. A description of how networks and expression data together with genetic data or GWAS findings can be used for the identification of genes/mutations associated with risk are reviewed by Bjorkegren et al. in relation to coronary artery disease (Bjorkegren et al., 2015). In similar approaches to those applied to tumor studies, gene expression data is used to infer co-expression networks. For networks showing correlation with phenotypic characteristics, possible regulatory genes are identified using Bayesian network algorithms. Alternatively, or complementarily, risk genotypes from GWAS are obtained and the networks are analyzed for enrichment of risk factors.

Gene Set Association Analysis (GSAA) is an integration tool developed by Xiong et al., for the identification of gene sets associated with disease (Xiong et al., 2012). In this setting, gene expression and SNV data are integrated to identify gene sets, defined by prior knowledge, enriched for disease associated gene expression and SNVs. GSAA calculates a disease association score per gene for each dataset and combines the two scores per gene. The scores are used to rank the resulting gene list, which is used to identify disease associated gene sets. The tool has been evaluated on both cancer and Crohn's disease-related gene sets from the Molecular Signatures Database (MSigDB). The tool is freely available with a version for gene expression array and one for RNAseq gene expression data.

Gene expression data can be seen as the functional link between SNVs and the disease they are associated with, as utilized by Huang et al. in the tool iGWAS (Huang et al., 2015). The tool incorporates knowledge from family-based association study designs and was applied to analyze genetic risk factors for asthma. iGWAS aims to specify the mechanism through which a mutation “causes” disease—such as through regulation of gene expression or alternative biological or environmental mechanism. Filtering of GWAS hits can be supported by information of the SNP's roles as eQTLs as this support a functional effect. However, with iGWAS a joint analysis of the two datasets is proposed, where SNPs and regulated genes are connected using knowledge of eQTLs and their targets. The tool therefore requires some level of prior knowledge.

A similar strategy has been published by Huang et al. for analyzing the total effect of SNP and gene expression on disease risk (Huang et al., 2014). This strategy can be used to analyze population based case control datasets, however is not developed to split the effect according to mechanism.

Hunang et al., have further proposed a model for integrated analysis of mQTL, eQTL, and GWAS to evaluate the combined effect of DNA methylation, gene expression and SNPs on disease risk (Huang, 2014). Both the effect of SNPs and DNA methylation on expression and the effect of SNPs on methylation is “integrated out” to further identify the effect directly assignable to the SNPs. The model is evaluated on risk for childhood asthma where 25 genes are identified compared to 5 for SNP-only analysis.

As with tumor-derived data, gene expression data is the dominating measure for functionality in risk integration studies. Of in-sequence variations, SNP array data for the analysis of SNV-sets appear to be commonly applied, leaving a gap for MPS based mutation analysis and CNV analysis. One reviewed strategy includes DNA methylation data in the integration, however other tools such as GSAA could be further developed to include this variation type. With the inclusion of gene expression and DNA methylation data in association studies it is important to consider that the variations are tissue specific and one must therefore be careful in interpreting their relationship to disease risk when data is not available for the affected tissue.

## Importance of data source and format in data integration

As mentioned above, the field of data integration in genome-wide cancer research has seen a shift from being dominated by array data (Yang et al., 2011; Neumann et al., 2012; Rakosy et al., 2013) to more often including data from MPS platforms. This development reflects both the history of platform development and financial considerations. As MPS platforms are becoming increasingly available and competitive in price to the array platform, MPS is more often the source of data including measurements of CNV and DNA methylation variation.

The bioinformatics process of obtaining information on CNVs and DNA methylation levels is different for each platform, however we will not touch further on this subject. As the format of data obtained from the two platforms most often is compatible when information on DNA methylation and gene expression is reached, most integration tools analyzing these variation types has no platform requirement. That is, the user must format the data to meet the requirement of the selected tool.

Raw gene expression and DNA methylation data can be analyzed to obtain information regarding the magnitude of an increase or decrease in gene expression or DNA methylation between cases and controls. The resulting list of variables is intuitively easier to shortlist, e.g., by selecting the highest absolute values, as compared to non-continuous variables such as SNPs and indels. It is therefore not the source but the type of variation that presents a challenge for the integration tools in these instances.

Information of direction and magnitude can be integrated and patterns identified e.g., for the stratification of tumor subtypes (Yang et al., 2011; Figueroa et al., 2013; Rhee et al., 2013). This integration can be applied to the full lists of identified variation limiting the requirement for pre-integration filtering. Therefore a number of methods and software programs have been developed for integrated analysis of variation types with information on direction and magnitude of differences. SIGMA2 (Chari et al., 2008) and InCroMAP (Wrzodek et al., 2013) are two of the newer tools. Both tools integrate data from different platforms by coordinates.

SIGMA2 is a tool for integrated analysis of cancer genomes (DNA copy number and allelic imbalance), epigenomes (DNA methylation and histone modification), and transcriptomes (mRNA and miRNA expression). Data from multiple platforms can be imported and analyzed, and the integrated data can be visualized simultaneously. SIGMA2 allows for identification of genetic locations affected by different types of variations, identification of genes whose expression is regulated by variations at the DNA level, and a number of additional integration-dependent analyses (Chari et al., 2008). The tool requires separate initial analysis of the different types of data. However, it allows for integrated analysis of the full datasets, thereby facilitating effective integration of large amounts of data without the risk of pre-integration loss of low effect variations.

Information on SNPs and indels (mutations) from MPS platforms is increasingly available, calling for the development of integration models suitable for these variations. However, dissimilar data formats and data types (continuous vs. discrete), together with dissimilar biological interpretation, render any integration of different types of variations non-trivial. A study by Mo et al. further developed the iCluser tool to iCluster+ to allow inclusion of discrete variables from sequencing data (Mo et al., 2013).

As seen in the above integration strategies, most studies implementing mutation data pre-analyze and filter the data to gene level to identify if a gene contains a mutation in a given sample or not. A high level of preprocessing is necessary when working with MPS data to reach this format together with more or less arbitrary cutoffs, which are applied to remove low quality calls and variations predicted to have low effect. This can have a big effect on the results of the integration as discussed below.

## Strength and weaknesses of strategies for data integration

### List intersection tests as a model for data integration

List intersection tests or versions hereof have been used for a number of integration projects analyzing data from tumor samples (Sadikovic et al., 2008, 2009; Ali Hassan et al., 2014). In short, the test analyses each dataset separately to obtain a list of genes containing the given variation. The intersection between the lists is then identified, containing genes affected by more than one type of variation. This approach limits the issues arising from cross-platform integration, because pre-processing, variation calling, and filtering is performed separately for each dataset. The lists of genes resulting from dataset-specific analysis are the subjects for further integration.

Directly intersecting lists of genes from genome-wide datasets will, in most instances, identify a high number of genes disrupted by more than one type of variation, however, the extent to which the genes are disrupted will vary greatly. For example, in the case of DNA methylation data, both differential methylation of a single CpG and clustering of highly differently methylated sites can disrupt the function of a gene (Weber et al., 2007; Bock, 2012; Jones, 2012). Integration projects that compare gene lists between studies using list-intersection models often address this issue by shortlisting genes according to a hypothesis of interest (e.g., predicted damaging effect of variations, extent of association and/or similarity to known phenotype-related genes). Following shortlisting, the new lists are compared and genes appearing in multiple lists are identified for further investigation.

Prior biological knowledge and interpretation of the functional relevance of a given gene are factors that can greatly influence the results with this approach. Prioritization tools are available that allow for automatic shortlisting of candidate genes by combining different types of information, such as sequence conservation, gene expression and linkage data. Such tools are becoming increasingly flexible and inclusive with respect to the extent of customization and the sources and types of information included in the prioritization. A number of prioritization tools also allow inclusion of different types of custom data such as gene expression, linkage and CNV data, thereby offering a level of late-stage integration in the process of selecting candidate genes.

Shortlisting of genes using a-priori defined information arguably limits the potency for novel findings. On the other hand, some level of shortlisting is necessary to limit the number of false positive findings. The level of shortlisting that is necessary depends on the goal of the study, but in general performing shortlisting of genes prior to data integration may reduce some of the advantages of having multiple types of data from the same set of samples.

### Structuring cross-platform data based on coordinates or higher-level patterns

Development of statistical methods for integrated analysis is challenged by the dissimilar biological implication of each type of variation, the different data formats, and the difference in number and genetic location of interrogated sites. Many integration methods have overcome these issues by performing platform-specific pre-processing and variation calling, followed by grouping of variations across platforms into variation sets according to their genetic location.

An important factor for this strategy is to decide how to group variations in the most optimal way. Often a group is defined on the basis of known genes. Through this approach, all variations located within the same gene are grouped, possibly including a predefined number of base pairs up and downstream from the target gene. An alternative strategy is to implement a sliding window with or without a degree of overlap. The definition of the set is important for the interpretation of the results, as the specific location of a variation, even within a gene, is important for its biological effect. For example, the effect a change in DNA methylation has on the expression of a gene is highly dependent on the genetic location of the methylation within the gene region. While hyper-methylation of a gene promoter is known to down-regulate the expression of the related gene, a change of DNA methylation within the gene body is believed to have alternative functions (Kulis et al., 2013).

By grouping variations according to coordinates it is anticipated that variations located at the same region exhibit similar effects on the phenotype or influence the same functional element. This fails to consider the complex interplay between functional elements and distant regulatory elements, such as trans QTLs, enhancers, inhibitors and TFBS.

### Functional evaluation supported by higher-level information

As an alternative to structuring data based on coordinates, a number of studies structure datasets based on higher-level patterns. Such patterns can be identified by implementing knowledge of functions, pathways and networks (e.g., from protein-protein interaction databases) or by directly identifying structures in the datasets. Identification of higher-level patterns in datasets is being explored for a range of applications, including data reduction, disease classification, and identification of disease markers and drug targets (Kutalik et al., 2008). Basing data integration on higher-level patterns allows researchers to include knowledge of complex structures in the human genome, and a high number of approaches are being evaluated for this type of integration (Kutalik et al., 2008; Mitra et al., 2013). Most studies integrate one type of data, often expression data (Sohler et al., 2004; Sivachenko et al., 2007; Qiu et al., 2010), with existing knowledge of higher-level patterns, but newer studies aim to overlay multiple datasets with patterns from online databases (Cancer Genome Atlas Research Network, 2013; Wen et al., 2013).

A number of tools that integrate data by generating variation sets are designed to take advantage of the known interaction between functional elements by implementing knowledge of such relations from online databases. This can facilitate the identification of a number of biologically relevant interactions. For example, it would be possible to identify the co-presence of a hypermethylated microRNA and an upregulated target gene. Furthermore, knowledge of shared functionalities of genetic locations can be used to evaluate patterns for identified disease relevant variations identified through integration studies, such as identified driver genes. For either approach, the source of pathway and network information and the strategy for analysis must be considered carefully.

Enrichment based methods such as gene set enrichment analysis (GSEA) and singular enrichment analysis (SEA) are popular for incorporating knowledge of pathways and networks or other predefined gene sets in analysis of heterogenic data or identified best hits. A number of online tools perform enrichment analysis for an applied list of genes by incorporating gene sets from online databases such as GO (http://www.geneontology.org) and KEGG (Kanehisa and Goto, 2000). A discussion of enrichment tools is found in Huang da et al. (2009).

PARADIGM (Vaske et al., 2010) is a method for pathway analysis of heterogenic data, which goes a step further by incorporating information on the type of interaction between elements of a pathway. It enables the analysis of low frequency variations in cross-platform datasets thereby supporting the analysis in situations where the disease in each person, or subgroup of people, is caused by different types of variations (for additional methods for integrative network analysis, see Cerami et al., 2010; Wu et al., 2010; Ciriello et al., 2012).

Creating a tool that incorporates biological knowledge in the analysis process is highly dependent on the availability of reliable, maintained, and well-structured databases. Much effort is being put into organizing knowledge of functional interactions into online databases. Yet, as knowledge of the genetic location and function of non-coding functional elements is far behind our understanding of coding genes, development of inclusive and well-structured databases will most likely remain the bottleneck for some time.

Using knowledge of pathways and networks to support integration of distant variations is affected by a similar range of challenges that are also affecting alternative integration approaches. These issues include the need for annotation, the limitation of our knowledge of functional elements, dependency on prior biological knowledge and the difficulty of handling discrete variables.

### Data reduction

Genome-wide datasets contain much information and it is a challenge to interpret the biological implication for any fraction of the observed variation. These datasets are often “noisy” as they contain a high number of low effect variations, common inter-individual variations, and a number of less reliable variations. When integrating the analysis of multiple data sets the complexity increases drastically. Therefore, methods for the integrated analysis of genome-wide data sets are required to reduce the complexity of the data. This reduction of complexity is particularly important for studies of disease risk, which have tended to search for *the* disease associated variation and therefore requires extensive shortlisting.

One approach is to effectively rank all identified variations according to a level of relevance. Variation in DNA methylation and gene expression levels can be ranked using the continuous values of direction and magnitude of methylation or expression, respectively. A number of approaches for ranking of these datasets and for the integrated analysis of ranked lists have been published (Boulesteix and Slawski, 2009; Kolde et al., 2012). In contrast, discrete values obtained when analyzing SNP and indels by sequencing platforms pose an issue, as the lack of a single continuous variable implies that there is no single platform-derived indication of effect size or disease relevance.

As an alternative, the ranking of in-sequence variations is often based on (i) a hypothesis of interest for the co-located gene, as seen with the list intersection model (in the form of shortlisting), or (ii) an estimate of effect size for the individual variation. A number of tools are available which can be implemented in the analysis of sequencing data to estimate the effect size of identified variations. Examples of such tools are Polyphen2 (Adzhubei et al., 2010) and SIFT (Kumar et al., 2009), which predict the effect of point mutations and indels identified by MPS on protein function.

Both ranking approaches employ a level of prior knowledge of the type of gene or variation that can cause disease. The implementation of prior knowledge can limit the strength to obtain novel findings, as discussed above. There is therefore a need for the development of more sophisticated strategies for ranking of genome-wide sequencing-based datasets. The MPS platform assigns a range of statistics to each called variation, which provides information on the reliability of the called variation. Furthermore, most analysis pipelines include software that adds information on population frequency and predicted damaging effects, as well as functional annotations to each called variation. This information allows consideration of the degree to which the variation is likely to be disease relevant. All of this information, or a subset of it, could form the basis for ranking of in-sequence variations from MPS platforms, however, this approach is associated with a number of biological and statistical challenges. The challenges include deciding what information to include in the ranking, to convert the information into a compatible scale for automatic ranking and in this process to decide to which extend each piece of information should influence the ranking.

An alternative approach to reduce the size or complexity of the datasets is through the identification of higher-level patterns as described above. This can be performed for each dataset independently or by an integrated approach (Kutalik et al., 2008). The integrated approach allows patterns present across the datasets to influence the data reduction thereby avoiding obliterating cross-dataset patterns prior to integration.

### Integrated analysis of non-coding variations

Data obtained from whole-genome platforms include inter-genic variations and variations located in non-coding genes. Integration approaches that focus on elements annotated during single-platform analysis are often restricted to working with well-documented elements (e.g., coding genes). However, knowledge of non-coding elements, their function, location and relevance, is growing rapidly, now making it important to include all identified variations when analyzing genome-wide datasets.

Information related to non-coding elements is available for download from several cost-free online sources such as the UCSC Genome Browser (http://genome.ucsc.edu). The UCSC table browser is an effective tool for downloading information on TF binding sites, Vista enhancers, conserved regions, ENCODE regulatory information, CpG islands, and lincRNA. In addition, data can be downloaded from a number of element-specific databases, and ENCODE data can be downloaded directly from the ENCODE database ftp access (ftp://ftp.sanger.ac.uk/pub/gencode/). This information, together with the freely available BEDtools (Quinlan and Hall, 2010) and SAMtools packages (Li et al., 2009), can be used to annotate lists of dissimilar variations based on genetic location, thereby obtaining a more comprehensive annotation of variation lists.

The ability to evaluate the function of identified non-coding elements is currently inferior to the ability to evaluate lists of coding elements, due to the different extent of knowledge available for the two types of elements. However, methods and tools such as motifbreakR are increasingly available, which can be used to predict effects of variants located in non-coding sequences such as enhancers or promoters (Chen et al., 2014; Coetzee et al., 2015). Such functional predictions can support the inclusion of non-coding variations in integration studies. The inclusion will most likely have greatest importance on integration studies that consider the functional interactions between variations at different genetic locations.

Notably, annotating variations with information about non-coding elements will not solve the issue of including inter-genic variations when the analysis models base the integration on co-location with functional elements. However, as information is obtained on the functional properties of a greater portion of the genome, fewer variations will be excluded from analysis.

The section on strength and weaknesses of strategies for data integration highlights the most dominating strategies including list intersection approaches and integration based on genetic co-ordinates or higher level patterns. The grouping of variations based on genetic location to facilitate e.g., a list intersection or enrichment based approach has been used to overcome the issues of dissimilarity between datasets from different platforms. However, the approach show limited abilities to consider the detailed functional differences related to the type of variation or precise genetic location, such as the location within the individual genes. There are further challenges in including non-coding and inter-genic variations in many existing integration strategies. For integration tools that include mutation data, there is a need for more sophisticated strategies for ranking and filtering to reduce false positives and identify the most disease associated variations or loci. Common for many integration strategies is also the use of a-priori information, for which both the limitations of current knowledge and the possible limitation for novel findings must be considered.

## Impact of integration on sample size

The possible increase in sensitivity for low frequency variations and highly heterogenic sites gained by integrated analysis may impact the sample size required for discovery of “causal” genetic events. Increased sensitivity toward low frequency variations means that one can extend the approach to situations involving rare samples, such as specimens from individuals with a rare cancer. A demand for large sample sizes would, in such situations, entail a broadening of selection criteria. This would in turn increase the risk that different disorders would be represented and thereby result in an increasingly heterogeneous group of underlying variations.

## Concluding remarks

Multiple types of variations, both genetic and epigenetic, are implicated in the development of complex disorders. It is therefore anticipated that integrated analyses could support the understanding of the risk factors and pathways of pathogenesis for these disorders. A number of studies have successfully performed integrated analysis of data from cancer samples, and have thereby obtained a more comprehensive understanding of the complex architecture of cancer genomes. However, many challenges remain and need to be solved before we can fully exploit integration of cross-platform data.

The process of analyzing MPS data to achieve information on disease-relevant SNPs and indels, include filtering based on measures of functional effect and scores of data quality. Reviewed integration strategies include MPS data after pre-processing, possibly missing some advantages of data integration. In order for data integration to increase sensitivity for genetic locations that are affected at low frequency in a single dataset or by low effect variations, the variations must not be lost prior to integration. The extensive level of filtering performed for whole-exome or whole-genome MPS data prior to integration entail the risk of misinterpretation and exclusion of relevant variations before data integration is performed.

Including measures of functional effect for identified variations obtained from software used to analyze MPS data could support development of integration strategies that have more complex definitions of function. Data on gene expression levels is dominating integration studies as a measure of functional effect. However, not all functionally important variations will affect gene expression, opening up for better integration of the tools developed for MPS data. These aspects suggest a need for more sophisticated and interconnected approaches to data filtering and integration, possibly in part by implementation cross-platform information in these steps.

While integration of extensively preprocessed datasets entail the risk of losing important variations prior to integration, it also presents the risk of including false positives in the integration. When integration is based on tables with binary numbers as seen for many of the described tools, little information is left regarding the quality of the variation. This leaves little room for evaluating the results of the integration. Again, better integration of the first steps of data analysis could be used to address this issue, but for now it is important to stress the need to critically evaluate the pre-integration process.

Current integration strategies tend to group variations according to genetic location. The grouping is often based on co-location or proximity to known genes, thereby excluding inter-genic variations from analysis. This limits the utility of current integration models for whole-genome datasets. Furthermore, strategies which group variations according to genetic location often fail to consider the connection between transacting or distant located regulatory elements and their targets. However, these methods allow for approximate identification of genes disrupted by different classes of variability such as highly heterogenic genes.

Large-scale data-sharing projects such as TCGA (http://cancergenome.nih.gov/) and ENCODE (Consortium et al., 2012) include multiple types of data on a range of phenotypes. The sharing of data and the growing interest in generating cross-platform datasets has enabled large-scale integrated analyses. Researchers working on these projects, together with groups who focus on the biostatistical and computational aspects, are leading the way in the creation of new integration strategies and tools. Existing integration tools illustrate a requirement for closer collaborations between the specialties so that knowledge of the MPS platforms can be implemented in the integration tools.

Despite the shortcomings of the current approaches, the results obtained so far indicate that integrated analysis of cross-platform datasets has the potential to provide new insights into the causes and pathogenesis of cancer.

## Author contributions

LT is the main author of the article. MS, MT, and LLH functioned as supervisors for LT and contributed with guidance to frame, content and structure. EM contributed with biostatistical guidance to content. LA contributed with knowledge of integration tools.

## Funding

MS is a National Health and Medical Research Council Senior Research Fellow (APP1061177). We have no additional funding to declare.

### Conflict of interest statement

The authors declare that the research was conducted in the absence of any commercial or financial relationships that could be construed as a potential conflict of interest.
